# Evaluation of 18 commercial serological assays for the detection of antibodies against SARS-CoV-2 in paired serum samples

**DOI:** 10.1007/s10096-021-04220-7

**Published:** 2021-03-17

**Authors:** Daniëlle A. T. Hanssen, Michiel Slaats, Marlies Mulder, Paul H. M. Savelkoul, Inge H. M. van Loo

**Affiliations:** 1 Department of Medical Microbiology, Maastricht University Medical Center Maastricht, PO 5800, 6202AZ Maastricht, The Netherlands; 2 Care and Primary Health Research Institute (CAPHRI), Maastricht University, PO 5800, 6202AZ Maastricht, Netherlands

**Keywords:** Antibody testing, ECLIA, ELISA, SARS-CoV-2, serology, POCT

## Abstract

A variety of serological tests have been developed to detect the presence of antibodies against the severe acute respiratory syndrome coronavirus 2 (SARS-CoV-2). We evaluated the performance of 18 commercially available SARS-CoV-2 antibody assays. Early (6–8 days after the start of symptoms) and late sera (>14 days) from ICU patients (*n*=10 and *n*=16, respectively) and healthcare workers (*n*=5 and *n*=9, respectively) were included. Additionally, 22 sera were included to detect potential cross-reactivity. Test characteristics were determined for the 18 assays. In >14 days samples, the Vircell IgG and Wantai Ig ELISAs had superior sensitivity compared to the other ELISAs (96%). Furthermore, the Roche Ig, the Epitope Diagnostics IgM, Wantai IgM, Euroimmun IgG, and IgA all showed a specificity of 100%. The POCTs of Boson Biotech and ACRO Biotech showed the highest sensitivities: 100% and 96% (83.5–99.8), respectively. The POCT of Orient Gene Biotech, VOMED Diagnostics, and Coris-Bioconcept showed highest specificities (100%). For the IgM and IgA assays, the Euroimmun IgA test showed the highest sensitivity in early samples: 46.7% (23.5–70.9) to 53.3% (29.1–76.5). In general, all tests performed better in patients with severe symptoms (ICU patients). We conclude that the Wantai Ig and Vircell IgG ELISAs may be suitable for diagnostic purposes. The IgM/IgA tests performed poorer than their IgG/Ig counterparts but may have a role in diagnoses of SARS-CoV-2 in a population in which the background seroprevalence of IgG high, and IgM and/or IgA may distinguish between acute or past infection.

**Supplementary Information:**

The online version contains supplementary material available at 10.1007/s10096-021-04220-7.

## Purpose

In December 2019, a novel coronavirus, called severe acute respiratory syndrome coronavirus 2 (SARS-CoV-2), emerged in Wuhan in the province of Hubei, China [1]. Infections due to SARS-CoV-2 can cause a wide variety of symptoms: from asymptomatic to mild to severe with a case fatality rate of 5% worldwide in confirmed cases according to WHO (https://www.who.int/emergencies/diseases/novel-coronavirus-2019).

In order to prevent further spread of the virus, worldwide, countries have implemented social distancing measures to prevent further spread of the virus. Many of these countries are struggling how and when to diminish these measures. Herein, serological testing can be a relevant tool. First, it adds in diagnosing in situations where PCR seems to be false-negative or when testing is performed after the acute phase of disease and the virus is already cleared. Second, serology can be helpful in contact tracing, and third, it can be used in epidemiological studies. Therefore, the society calls for serological testing to determine who has already been infected by SARS-CoV-2 and who has not, hoping that knowing the infection rate can help in preventing resurgences. For the majority of the population, it is still unknown whether they already have been infected by SARS-CoV-2 due to limitations in test availability and the possibility of asymptomatic infections. Consequently, well-validated serological assays are essential.

Many manufacturers offer serologic tests, but reports suggest that the sensitivity and specificity of these tests vary, especially in individuals with mild symptoms [2, 3]. Hence, we evaluated the performance of 18 commercially available assays in a reference panel of sera from patients with mild to moderate and severe symptoms.

## Methods

### Study population

The reference panel included sera from 16 patients admitted to the ICU of the Maastricht University Medical Center (MUMC+) in March and April 2020 and 9 healthcare workers (HCWs) from the MUMC+. The ICU patients were all classified as severe cases. The HCWs were classified as asymptomatic or having mild (cough, running nose, sore throat, diarrhea, headache) or moderate symptoms (fever, dyspnea). None of the HCWs was admitted to the hospital. In all individuals, an infection with SARS-CoV-2 was confirmed by PCR. The first date of symptoms according to the medical file (in case of the ICU patients), or according to a questionnaire (in case of the HCWs), was obtained. We collected early sera (6 to 8 days after onset of symptoms) and late sera (>14 days after onset of symptoms). A late serum was available for the complete reference panel (16 ICU patients and 9 HCWs). A corresponding early serum of the same individual was available in 10 ICU patients and 5 HCWs.

In addition, 22 sera from patient samples were included to determine specificity. Of these samples, 12 were randomly selected from patients before December 2019 and included men and women in different age groups (range 6 to 78). These sera were collected by various specialists, including general practitioners, hematologists, cardiologists, neurologists, and fertility specialists, and the reason for serum collection was diverse: diagnostic workup screenings for patients with neurologic symptoms, mononucleosis-like illness, endocarditis, screenings prior to fertility treatments, and pre-transplantation screenings in hematologic patients. None of the individuals was diagnosed with an infection that could possibly cause cross-reactivity, as described below. For detection of cross-reactivity, we included samples from patients with characteristics or infections which are known to have a higher likelihood of false-positive test results: 1 pregnant woman, 2 samples of patients diagnosed with an acute Epstein Barr virus infection (EBV), 3 patients diagnosed with an acute *Mycoplasma pneumoniae* infection, and 4 patients recently diagnosed with an infection with another coronavirus (229E, OC43 and NL63). These samples were also collected before December 2019.

The local institutional review board, the Medical Ethical Committee, of the Maastricht UMC+ approved the study, which will be performed based on the regulations of Helsinki. During the pandemic, the board of directors of Maastricht UMC+ adopted a policy to inform patients and ask their consent to use the collected data and stored leftover serum samples for COVID-19 research purposes.

### Diagnostic tests

All sera were tested with the following nine ELISAs: EDI Novel Coronavirus COVID-19 IgG and IgM ELISA kit (Epitope Diagnostics, Inc., Hannover, Germany), Anti-SARS-CoV-2 ELISA IgG and IgA (Euroimmun, Lübeck, Germany), *recom*Well SARS-CoV-2 IgG (Mikrogen Diagnostik, Neuried, Germany), COVID-19 ELISA IgG and IgM/IgA (Vircell, Selinion, Granada, Spain), and Wantai SARS-CoV-2 Ab and IgM ELISA (Beijng Wantai Biological Pharmacy Enterprise Co., Ltd., Beijing, China). All ELISAs were performed on the Virion\Serion Immunomat (Virion\Serion, Würzburg, Germany). Additionally, the sera were tested using the ElectroChemiLuminescence ImmunoAssay (ECLIA) Elecsys Anti-SARS-CoV-2 Ig (Roche Diagnostics GmbH, Mannheim, Germany) using the Cobas 8000 (Roche Diagnostics GmbH, Mannheim, Germany).

Eight point-of-care tests (POCT) were evaluated: 2019-nCoV IgG/IgM Rapid Test Cassette (ACRO Biotech Inc, Rancho Cucamonga, USA); Rapid 2019-nCoV IgG/IgM Combo Test Card (Boson Biotech Co., Ltd, Xiamen, China); Medea Medical COVID-19 rapid test (Medea Medical Co., Nootdorp, The Netherlands); COVID-19 IgG/IgM Rapid Test Cassette (Orient Gene Biotech Co., Ltd, Zhejiang, China); VivaDiag COVID-19 IgM/IgG Rapid Test (VivaChek Laboratories, Inc., Wilmington, USA); COVID-19 IgG/IgM Rapid Test Cassette (VOMED diagnostics, Vega Medicare, Telangana, India); COVID-19 IgG/IgM Rapid Test (CTK Biotech, San Diego County, USA); and Coris-Bioconcept COVID-19 Sero NP/RBD (Coris-Bioconcept, Gembloux, Belgium). Any visible line (weak or strong) in either the IgG or IgM or both was considered to be positive. All tests were performed and interpreted according to the manufacturers’ instructions.

Due to a limited availability of tests, only a small subset of samples (*n*=20) was tested with the POCT from VivaChek Laboratories, whereas a somewhat larger subset was tested with the POCT from Boson Biotech, ACRO Biotech, and Coris-Bioconcept (*n*=43, *n*=61, and *n*=60, respectively). The other POCTs of VOMED Diagnostics, Orient Gene Biotech, Medea Medical, and CTK Biotech were used on all samples included.

### Analysis

All analyses were performed using SPSS 25 (IBM). Sensitivity, specificity, and 95% CI were calculated for each test. Separate analyses were performed for 6 to 8 days and >14 days samples and for severe and mild to moderate cases. As it is yet unclear how to interpret the borderline results, we used two calculations. The first one with borderline values counted as positive and the second with borderline values counted as negative, resulting in a range of different test characteristics. To assess the clinical accuracy of the different ELISAs/ECLIA, receiver operating characteristics (ROC) curves were done to calculate the area under the curve (AUC) [4].

## Results

### ELISA/ECLIA

We compared the sensitivity of ELISA/ECLIA kits of six different manufacturers, all of which had an Ig or IgG test, whereas four manufacturers also had a separate IgM, IgA, or combined IgM/IgA test (Table 1). The sensitivity of the >14 days samples was > 95% for Vircell IgG and Wantai Ig (96.0%, 83.5–99.8). Sensitivity was <95% for the tests of Euroimmun IgG (92%, 77.3–98.6), Mikrogen Diagnostik IgG (ranging from 88, 71.8–96.9, to 92%, 77.3–98.6), Epitope Diagnostics IgG (88%, 71.8–96.9), and the Roche Ig (88.0%, 71.8–96.9). In all cases, the sensitivity of the IgA/IgM tests was similar or lower than the sensitivity of the Ig/IgG test of the same manufacturer in the >14 days samples (Vircell IgM/IgA ranging from 88.0, 71.8–96.9, to 96.0%, 83.5–99.8; Euroimmun IgA 92.0%, 77.3–98.6; Wantai IgM 88.0%, 71.8–96.9; and Epitope Diagnostics ranging from 64.0, 44.5–80.8, to 80.0%, 61.8–92.3). For Vircell IgG, sensitivity ranged from 46.7, 23.5–70.9, to 60%, 35.1–81.7, and for Wantai Ig, this was 53.3%, 29.1–76.5, in the 6 to 8 day samples. For the IgM and IgA assays, Euroimmun IgA test showed highest sensitivity (ranging from 46.7, 23.5–70.9, to 53.3%, 29.1–76.5) (Table 1).

**Table 1:** Performances of ELISAs/ECLIA for SARS-CoV-2 antibody detection

	Early samples			Late samples				
	Sensitivity overall in % (95% CI) (*n*=15)	Sensitivity ICU in % (95% CI) (*n*=10)	Sensitivity HCW in % (95% CI) (*n*=5)	Sensitivity overall in % (95% CI) (*n*=25)	Sensitivity ICU in % (95% CI) (*n*=16)	Sensitivity HCW in % (95% CI) (*n*=9)	Specificity in % (95% CI) (*n* = 22)	AUC (95% CI)
ELISA								
Epitope Diagnostics IgG	33.3 (13.5–58.5)	40.0 (14.6–70.0)	20.0 (1.3–62.8)	88.0 (71.8–96.9)	100	66.7 (34.5–90.5)	95.5 (81.5–99.7)	0.97 (0.93–1.00)
Epitope Diagnostics IgM	26.7 (9.2–51.5)–33.3 (13.5–58.5)^a^	30.0 (8.5–60.7)–40.0 (14.6–70.0)^a^	20.0 (1.3–62.8)	64.0 (44.5–80.8)–80.0 (61.8–92.3)^a^	87.5 (66.2–97.8)–93.8 (75.3–99.6)^a^	22.2 (4.1–54.2)–55.6 (24.9–83.5)^a^	100	0.95 (0.89–1.00)
Euroimmun IgG	6.7 (0.4–26.2)–13.3 (2.3–35.8)^a^	10.0 (0.6–37.2)–20.0 (3.6–49.9)^a^	0	92.0 (77.3–98.6)	100	77.8 (45.8–95.9)	100	0.97 (0.91–1.00)
Euroimmun IgA	46.7 (23.5–70.9)–53.3(29.1–76.5)^a^	60.0 (30.0–85.4)	20.0 (1.3–62.8)–40.0 (8.1–80.1)^a^	92.0 (77.3–98.6)	100	77.8 (45.8–95.9)	100	0.97 (0.91–1.00)
Mikrogen Diagnostik IgG	26.7 (9.2–51.5)	30.0 (8.5–60.7)	20.0 (1.3–62.8)	88.0 (71.8–96.9)–92.0 (77.3–98.6)^a^	100	66.7 (34.5–90.5)–77.8 (45.8–95.9)^a^	95.5 (81.5–99.7)	0.95 (0.88–1.00)
Vircell IgG	46.7 (23.5–70.9)–60.0(35.1–81.7)^a^	60.0 (30.0–85.4)–70.0 (39.3–91.5)^a^	20.0 (1.3–62.8)–40.0 (8.1–80.1)^a^	96.0 (83.5–99.8)	100	88.9 (59.5-99.3)	95.5 (81.5–99.7)	0.99 (0.96–1.00)
Vircell IgM + IgA	40.0 (18.3–64.9)	50.0 (21.8–78.2)	20.0 (1.3–62.8)	88.0 (71.8–96.9)–96.0 (83.5–99.8)^a^	100	66.7 (34.5–90.5)–88.9 (59.5–99.3)^a^	68.2 (47.5–84.9)–77.3 (57.4–91.2)^a^	0.92 (0.84–1.00)
Wantai Ig	53.3 (29.1–76.5)	60.0 (30.0–85.4)	40.0 (8.1–80.1)	96.0 (83.5–99.8)	100	88.9 (59.5–99.3)	95.5 (81.5-99.7)	0.98 (0.94–1.00)
Wantai IgM	40.0 (18.3–64.9)	50.0 (21.8–78.2)	20.0 (1.3–62.8)	88.0 (71.8–96.9)	100	66.7 (34.5–90.5)	100	0.96 (0.89–1.00)
ECLIA								
Roche Ig	33.3 (13.5–58.5)	40.0 (14.6–70.0)	20.0 (1.3–62.8)	88.0 (71.8–96.9)	100	66.7 (34.5–90.5)	100	0.99 (0.96–1.00)

*AUC* area under (ROC) curve, *ELISA* enzyme-linked immunosorbent assay, *ECLIA* electrochemiluminescence immunoassay, *Ig* immunoglobulin, *ICU* intensive care unit, *HCW* healthcare worker

Sensitivity, specificity and AUC for all ELISAs/ECLIA tested, using 25 late sera and 15 early sera of patients with a positive PCR for SARS-CoV-2 and 22 negative controls. 95% CI are shown in parentheses. The early samples include the samples 6 to 8 days after start of disease, whereas the late samples include the samples >14 days after start of disease

^a^Two calculations were used for borderline results: the first one with borderline values counted as negative and the second with borderline values counted as positive

For all tests, the sensitivity in sera from patients with severe disease was higher than in sera from patients with mild to moderate disease. Apart from the IgM test of Epitope Diagnostics (ranging from 87.5, 66.2–97.8, to 93.8%, 75.3–99.6), the sensitivity of all tests for the >14 days sera from patients with severe disease was 100%. For patients with mild to moderate disease, the sensitivity in the >14 days sera was highest for Vircell IgG and Wantai Ig (88.9%, 59.5–99.3). For the IgM and/or IgA tests, Vircell IgM/IgA (ranging from 66.7, 34.5–90.5, to 88.9%, 59.5–99.3) and Wantai IgM (66.7%, 34.5–90.5) showed highest sensitivity. Furthermore, for all tests, the sensitivity in 6 to 8 day sera was better for the sera from patients with severe disease compared to sera from patients with mild to moderate disease (Table 1).

For Roche Ig, Euroimmun IgG and IgA, Epitope Diagnostics IgM, and Wantai IgM, a specificity higher than 98% was found. The specificity of Epitope IgG, Mikrogen Diagnostik IgG, Vircell IgG, and Wantai Ig was 95.5%, 81.5–99.7, whereas the specificity of the Vircell IgM/IgA was least (ranging from 68.2, 47.5–84.9, to 77.3%, 57.4–91.2) (Table 2 and Table 4). The specificity was impaired due to cross-reactivity with EBV for Epitope IgG, Mikrogen IgG, Wantai Ig, and Vircell IgM/IgA, with *Mycoplasma pneumoniae* for Vircell IgG and IgM/IgA and with other coronaviruses (NL63 and 229E) for Vircell IgM/IgA.

**Table 2:** Technical characteristics of the different commercial tests

Manufacturer	Antibody target	Method	Operational type	Material	Automated	Serum volume	Time to result^a^	Comments
Epitope Diagnostics	IgG	ELISA	B	S	ELISA platform	10 μL	80 min	
Epitope Diagnostics	IgM	ELISA	B	S	ELISA platform	10 μL	80 min	
Euroimmun	IgG	ELISA	B	S, P	ELISA platform	10 μL	120 min	
Euroimmun	IgA	ELISA	B	S, P	ELISA platform	10 μL	120 min	
Mikrogen Diagnostik	IgG	ELISA	B	S, P	ELISA platform	10 μL	120 min	
Vircell	IgG	ELISA	B	S, P	ELISA platform	5 μL	95 min	30-min sample inactivation at 56°C
Vircell	IgM + IgA	ELISA	B	S, P	ELISA platform	5 μL	95 min	30-min sample inactivation at 56°C
Wantai	Ig	ELISA	B	S, P	ELISA platform	100 μL	75 min	
Wantai	IgM	ELISA	B	S, P	ELISA platform	10 μL	75 min	
Roche	Ig	ECLIA	RA	S, P	Cobas e 411, e 601, e 602 (Roche diagnostics)	20 μL	18 min	Death volume of approximately 150 μL
ACRO Biotech	IgG + IgM	POCT	M	S, P, WB	No	10 μL S/P; 20 μL WB	10 min	
Boson Biotech	IgG + IgM	POCT	M	S, P, WB	No	2 μL	15 min	
Medea Medical	IgG + IgM	POCT	M	S, P, WB	No	5 μL	10 min	
Orient Gene Biotech	IgG + IgM	POCT	M	S, P, WB	No	5 μL S/P; 10 μL WB	10 min	
VivaChek Laboratories	IgG + IgM	POCT	M	S, P, WB	No	10 μL	15 min	
VOMED Diagnostics	IgG + IgM	POCT	M	S, P, WB	No	10 μL	10 min	
CTK Biotech	IgG + IgM	POCT	M	S, P, WB	No	10 μL	15 min	
Coris-Bioconcept	NP + RBD	POCT	M	S, P	No	30 μL	15 min	

*ELISA* enzyme-linked immunosorbent assays, *ECLIA* electrochemiluminescence immunoassay, *POCT* point-of-care test, *B* batch, *RA* continuous random access, *M* manual, *S* serum, *P* plasma, *WB* whole blood, *NP* nucleocapsid protein, *RBD* receptor binding protein

^a^Duration of test only includes the time necessary for the different incubation steps; it does not include sample handling time and washing steps, which will be comparable for the different tests and be dependent of the number of samples

ROC curves of each test show an AUC higher than 0.95 for the Ig/IgG assays and higher than 0.92 for the IgM and/or IgA assays.

### POCT

Sensitivities higher than 95% were found for the POCT from ACRO Biotech (96%, *n*=27), Boson Biotech (100%, *n*=18), and VivaChek Laboratories (100%, *n*=9). The POCTs of Orient Gene Biotech (*n*=22), VivaChek Laboratories (*n*=10), and VOMED Diagnostics (*n*=22) showed the highest specificity (100%).

Cross-reactivity in sera from patients with EBV, other coronavirus infections (HCoV 229E), *Mycoplasma pneumoniae*, or a specific reactivity due to pregnancy was seen in the POCTs from ACRO Biotech, BOSON Biotech, and Medea Medical (Table 3 and Table 4).

**Table 3:** Performance of POCTs for SARS-CoV-2 antibody detection

	Early samples			Late samples			
	Sensitivity overall in % (95% CI) (*n*=15)	Sensitivity ICU in % (95% CI) (*n*=10)	Sensitivity HCW in % (95% CI) (*n*=5)	Sensitivity overall in % (95% CI) (*n*=25)	Sensitivity ICU in % (95% CI) (*n*=16)	Sensitivity HCW in % (95% CI) (*n*=9)	Specificity in % (95% CI) (*n*=22)
POCT							
ACRO Biotech	60.0 (35.1–81.7)	80.0 (50.1–96.4)	20.0 (1.3–62.8)	96.0 (83.5–99.8)	100	88.9 (59.5–99.3)	81.0 (61.1–93.7) (*n*=21)
Boson Biotech^a^	77.8 (45.8–95.9) (*n*=9)	100 (*n*=4)	60.0 (19.9–91.9) (*n*=5)	100 (*n*=18)	100 (*n*=13)	100 (*n*=5)	85.7 (62.1–97.5) (*n*=16)
Medea Medical	26.7 (9.2–51.5)	30.0 (8.5–60.7)	20.0 (1.3–62.8)	92.0 (77.3–98.6)	100	77.8 (45.8–95.9)	90.9 (74.5–98.4)
Orient Gene Biotech	46.7 (23.5–70.9)	50.0 (21.8–78.2)	40.0 (8.1–80.1)	92.0 (77.3–98.6)	100	77.8 (45.8–95.9)	100
VivaChek Laboratories^a^	0 (*n*=1)	-	0 (*n*=1)	100 (*n*=9)	100 (*n*=8)	100 (*n*=1)	100 (*n*=10)
VOMED Diagnostics	40.0 (18.3–64.9)	50.0 (21.8–78.2)	20.0 (1.3–62.8)	90.9 (74.5–98.4)	100	77.8 (45.8–95.9)	100
CTK Biotech	33.0 (13.5–58.5)	40.0 (14.6–70.0)	20.0 (1.3–62.8)	92.0 (77.3–98.6)	100	77.8 (45.8–95.9)	95.5 (81.5–99.7)
Coris-Bioconcept^a^	30.8 (10.7–57.7) (*n*=13)	44.0 (16.5–75.1) (*n*=9)	0 (*n*=4)	88.0 (71.8–96.9)	100	66.7 (34.5–90.5)	100

*ICU* intensive care unit, *HCW* healthcare worker, *POCT* point of care test

Sensitivity and specificity for POCTs tested, using 25 late sera and 15 early sera of patients with a positive PCR for SARS-CoV-2 and 22 negative controls. 95% CI are shown in parentheses

^a^Of these POCTs, only a subset of samples were tested, and amount of samples tested is noted after percentage. The early samples include the samples 6 to 8 days after start of disease, whereas the late samples include the samples > 14 days after start of disease

**Table 4:** Overview of cross reactivity of ELISA’s/ECLIA and POCTs in negative control sera, serum from a pregnant woman, and sera from patients diagnosed with other coronaviruses, acute EBV, and *Mycoplasma pneumoniae*

	False-positive result in seronegative sera^a^	Cross reactivity with other coronaviruses	Cross reactivity with acute EBV	Cross reactivity with *Mycoplasma pneumoniae*	Cross reactivity with pregnancy
ELISA/ECLIA					
Epitope Diagnostics IgG	0/12	0/4	1/2	0/3	0/1
Epitope Diagnostics IgM	0/12	0/4	0/2	0/3	0/1
Euroimmun IgG	0/12	0/4	0/2	0/3	0/1
Euroimmun IgA	0/12	0/4	0/2	0/3	0/1
Mikrogen Diagnostik IgG	0/12	0/4	1/2	0/3	0/1
Wantai Ig	0/12	0/4	1/2	0/3	0/1
Wantai IgM	0/12	0/4	0/2	0/3	0/1
Vircell IgG	0/12	0/4	0/2	1/3	0/1
Vircell IgM + IgA	1/12	1/4 (Coronavirus 229E)	2/2	2/3	1/1
Roche Ig	0/12	0/4	0/2	0/3	0/1
POCT					
ACRO Biotech	1/11	1/4 (Coronavirus 229E)	1/2	1/3	0/1
Boson Biotech	1/11	1/2 (Coronavirus 229E)	1/1	0/1	1/1
Medea Medical	0/12	0/4	1/2	0/3	1/1
Orient Gene Biotech	0/12	0/4	0/2	0/3	0/1
Vivachek Laboratories	0/6	0/2	0/1	NA	0/1
Vomed	0/12	0/4	0/2	0/3	0/1
CTK Biotech	0/12	0/4	0/2	0/3	0/1
Coris-Bioconcept	0/12	0/4	0/2	0/3	0/1

*ELISA* enzyme-linked immunosorbent assay, *ECLIA* electrochemiluminescence immunoassay, *Ig* immunoglobulin, *NA* not analyzed

All samples were collected before December 2019

^a^These sera were collected by various specialists (general practitioners, hematologists, cardiologists, neurologists, fertility specialists)

### Technical characteristics

The technical characteristics, concerning the material that can be used (plasma, serum, whole blood), input volume, turnaround time, target, and detection limit, are shown in Table 2.

In terms of ease of use, most ELISAs were similar, except for the Vircell tests. These tests need a serum inactivation step which makes it more difficult to incorporate the tests in an automated workflow. On the contrary, the ECLIA from Roche showed significant shorter time to results compared to the ELISAs and was easy to perform due to the fact that this test was performed on the random access system COBAS 8000. Notably, the test from Roche consists only of total Ig and needs a larger death volume and thus more serum.

The POCTs were all easy to perform but included more actions per test for the technicians. Difficulties in interpreting bands were experienced for the tests of ACRO Biotech, Medea Medical, Xiamen Boson, and Coris-Bioconcept. In these tests, the strength of the bands could be very weak, resulting in inconsistent interpretations between the two performing technicians.

## Conclusion

In this study, we compared the performance of 18 commercial assays for the detection of antibodies against SARS-CoV-2. Of the ELISAs tested, the Vircell IgG and Wantai Ig showed the highest sensitivity in both the >14 days sera (both 96%) and 6 to 8 day sera (46.7–60.0% and 53.3%, respectively). Specificity was 100% for Roche Ig, Euroimmun (IgG and IgA), Epitope Diagnostics IgM, and Wantai IgM. Although our study included a limited amount of samples, our observations correspond to similar high sensitivity and specificity percentages found for the Wantai Ig, IgM, Euroimmun IgG, and Euroimmun IgA by others [5–10]. Concerning the POCTs, sensitivity was >95% for ACRO Biotech and VivaChek Laboratories. Whitman et al. included around 10–19 patients in their analyses and reported a sensitivity for the POCT of VivaChek Laboratories of 78.9, 54.4–93.9, to 90.0%, 55.5–99.7 [9]. Contrastively to the results of a systematic review including 40 articles performed by Lisboa Bastos et al., we found higher sensitivities in the POCTs and ELISAs [11]. Lisboa Bastos et al. reported consistently lower pooled sensitivities for POCTs (66%, 49.3–79.3) compared to ELISAs (84.3%, 75.6–90.9) and ECLIAs (97.8%, 46.2–100). However, it is not shown which manufacturers are included. In our study, specificity was >95% for the tests from VOMED Diagnostics, Orient Gene Biotech, and VivaChek Laboratories. Specificity was least for the POCT of ACRO biotech due to cross-reactivity with coronavirus 229E and acute EBV. Cross-reactivity of the POCT of ACRO biotech with coronavirus HKU1 has been described previously [5].

In general, the detection of antibodies was better in sera taken >14 days after onset of disease. This can be explained due to the fact that the development of antibodies to SARS-CoV-2 can take up to 14–21 days [11, 12]. Additionally, in our study, detection of antibodies was higher in sera of individuals with more severe disease which is also observed by others [2, 6, 13]. Thus, an overrepresentation of samples from severely ill patients included in our study may have contributed to the higher overall sensitivities found.

Depending on the purpose for serological testing (prevalence screening versus diagnosing individual patients), specific test characteristics are more important than others [14]. In general, sensitivity higher than 95% and specificity higher 98% are required test characteristics to use serological tests for diagnostic purposes as well as for screening purposes. For diagnostic purposes in individual patients, high sensitivity is most important, while reflex testing in certain cases detecting false-positive results can overcome a somewhat lower specificity. For seroprevalence studies, a high specificity is preferred to minimize false-positive results. This increases the PPV, while the NPV is decreased minimally [15]. Especially in a population with low prevalence of SARS-CoV-2, high specificity is required in order to minimalize an overestimation of the true prevalence. On the other hand, tests with higher sensitivity are preferred if the pretest probability of the disease is higher, like in diagnosing individual patients. The Vircell IgG and Wantai Ig meet the criterion of high sensitivity but have a specificity lower than 98%. Even though we found a lower specificity, in our opinion, these are tests of choice for patient diagnosis, and to perform seroprevalence studies. Due to the overrepresentation of samples with other infections (EBV 2/22 and *Mycoplasma pneumoniae* 3/22*)* which have a higher likelihood to give false-positive test results, compared to the incidence of these infections in the general population at any given time, the specificity found in this study is probably an underestimation [16]. Therefore, the impact of cross-reactivity on specificity in the general population will be lower than found in our results and specificity will be higher.

In addition to the observed cross-reactions in the Wantai Ig and Vircell IgG, we also observed cross-reactivity in the Epitope IgG, Mikrogen IgG, and Vircell IgM/IgA due to other infections such as EBV, *Mycoplasma pneumoniae*, and other coronaviruses. Cross-reactions in the ELISAs of Epitope and Euroimmun ELISAs have been described previously in samples with antibodies to HCoV OC43, *Chlamydia pneumoniae*, respiratory syncytial virus, and *Streptococcus pneumoniae*; the latter three were not included in our tested samples [17, 18]. Although we did not observe cross-reactivity of the Euroimmun IgG ELISA, we cannot exclude possible cross-reactivity with coronavirus HKU1, as described by Lassaunière et al. [5]. The results on cross-reactivity do not necessarily affect population screening, since these infections are not highly prevalent [16]. However, individual patient cases test results may require reflex testing.

Although the performance in sensitivity and specificity of some ELISAs was quite similar, the individual sera showed inconsistency in results depending on which test was performed. These differences may be explained by the choice and concentration of SARS-CoV-2 antigen target [5]. Unfortunately, most tests do not give information about antigen target and detection limit. For example, the lower sensitivity of the Roche Ig test may be explained by the manufacturers’ statement that the test is mainly positive in individuals with neutralizing antibodies, which potentially could lead to an underestimating of the number of infected individuals. The Roche Ig uses a recombinant protein representing the nucleocapsid (N) protein, whereas most other assays use the receptor binding domain of the surface (S) protein [19].

Regardless of sensitivity and specificity, timing of antibody testing is crucial. Because of the delay in antibody response in the early stage of disease, PCR remains the test of choice within 10–14 days after onset of symptoms. As such, the lower observed sensitivities of the serologic tests in 6 to 8 day samples are not necessarily a result of a low sensitivity of the tests themselves but reflect an inadequate timing for antibody testing. However, when comparing the sensitivities of the different tests at one time point, regardless of the time between first day of symptoms and test date, an overall higher sensitivity of ELISAs which detect IgG or total Ig was observed in comparison to ELISAs which detect IgM/IgA [5, 6]. As IgM and IgA decrease after approximately 30 to 90 days [20], ELISAs focusing on IgM/IgA might be of special importance to diagnose SARS-CoV-2 infections in the future, when in a population the background seroprevalence of IgG is higher, and IgM and/or IgA may distinguish between acute or past infection.

Concerning the POCTs, variable results were observed. For example, the sensitivity of the POCTs from ACRO Biotech and Boson Biotech was comparable and for the early samples even better than the sensitivity of the ELISAs and ECLIA. However, the higher sensitivity impairs the specificity, which is less than 90%, mainly due to cross-reactivity with sera from patients who recently suffered from coronavirus 229E or EBV. Cross-reactivity of the POCT of ACRO biotech has been described in previous executed studies in samples of patients with EBV and HCoV OC43 [18]. Using the criteria of higher than 95% sensitivity and specificity the POCT of VivaChek Laboratories showed good results, with the limitation that the reference sera set could not be completely tested due to the limited number of tests available for evaluation.

The major limitation of this study is the limited number of samples that are used. Therefore, the conclusions from this study must be carefully interpreted. However, we think that this limitation is counterbalanced by the large number of tests compared on the same samples to make direct comparisons between the tests possible.

In conclusion, we compared several commercial assays for the detection of SARS-CoV-2 antibodies. We conclude that the Wantai Ig and Vircell IgG ELISAs may be suitable for individual patient diagnosis and also screening situations in low seroprevalence populations. The IgM/IgA tests performed poorer than their IgG/Ig counterparts.

The call for serological tests to detect antibodies against the novel SARS-CoV-2 virus is increasing. Antibody detection with the use of ELISAs was shown to correlate strongly with virus neutralizing antibodies using a PRNT50 assay [21]. However, more knowledge on the contribution of neutralizing antibodies to protection against future infections is needed. A study that investigated antibody response and immunity to the well-known seasonal coronaviruses HCoV-NL63, HCoV-229E, HCoV-OC43, and HCoV-HKU1 has shown substantial reduction of antibodies as soon as 6 months post-infection and frequent reinfections 12 months post-infection [22]. Moreover, there is much debate on the possibility of waning immunity, with a growing amount of evidence of a decline in neutralizing antibodies [20, 23, 24]. Future research on kinetics of antibody responses to SARS-CoV-2 is therefore needed to assess added value of serology in diagnosing SARS-CoV-2 in the future.

## Supplementary Information

ESM 1 (PNG 14 kb)

ESM 2 (PNG 14 kb)

ESM 3 (PNG 12 kb)

ESM 4 (PNG 14 kb)

ESM 5 (PNG 12 kb)

ESM 6 (PNG 14 kb)

ESM 7 (PNG 12 kb)

ESM 8 (PNG 12 kb)

ESM 9 (PNG 12 kb)

ESM 10 (PNG 12 kb)

## Data Availability

The datasets generated and/or analyzed during the current study are available from the corresponding author on reasonable request.
