# Supplementary figures and images for: Evaluation of 18 commercial serological assays for the detection of antibodies against SARS-CoV-2 in paired serum samples
Source: Eur J Clin Microbiol Infect Dis. 2021 Mar 17;40(8):1695–703. doi: 10.1007/s10096-021-04220-7 (PMC7968571; doi:10.1007/s10096-021-04220-7)

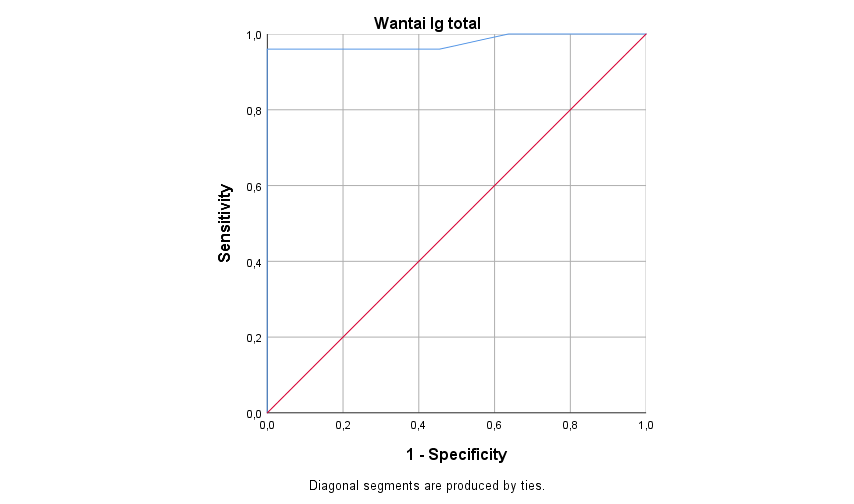

Supplement: Supplementary file 1 — (PNG 14 kb) [file 10096_2021_4220_MOESM1_ESM.png]

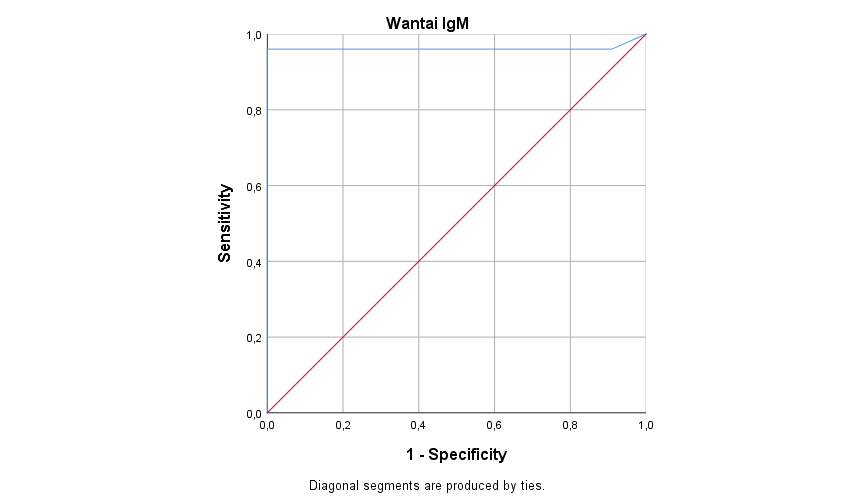

Supplement: Supplementary file 2 — (PNG 14 kb) [file 10096_2021_4220_MOESM2_ESM.png]

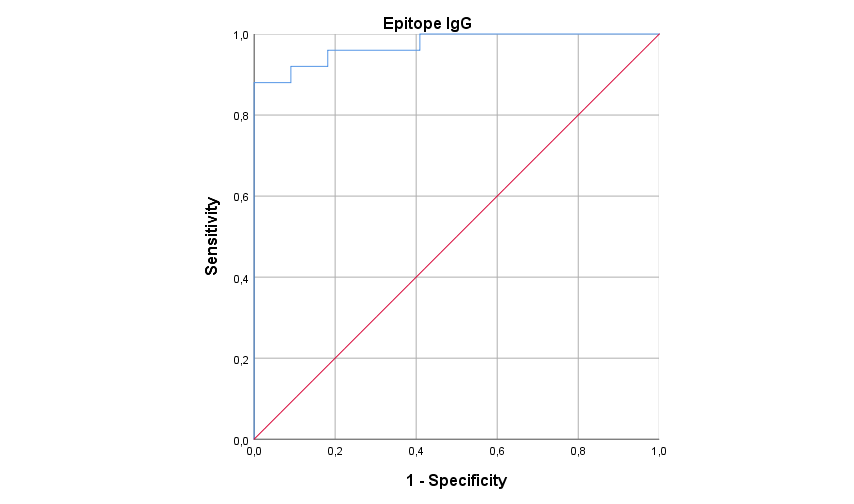

Supplement: Supplementary file 3 — (PNG 12 kb) [file 10096_2021_4220_MOESM3_ESM.png]

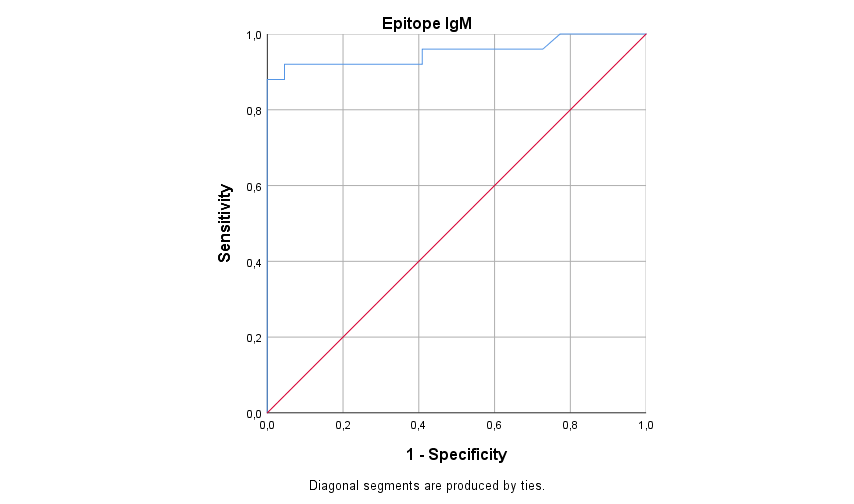

Supplement: Supplementary file 4 — (PNG 14 kb) [file 10096_2021_4220_MOESM4_ESM.png]

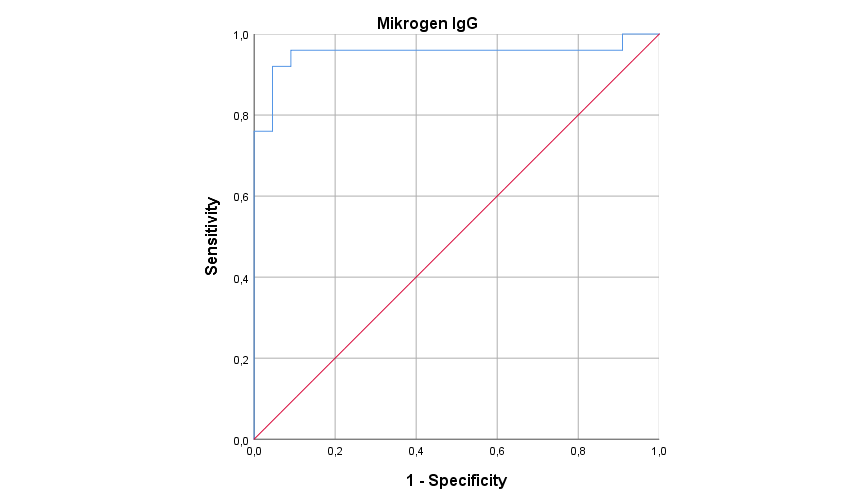

Supplement: Supplementary file 5 — (PNG 12 kb) [file 10096_2021_4220_MOESM5_ESM.png]

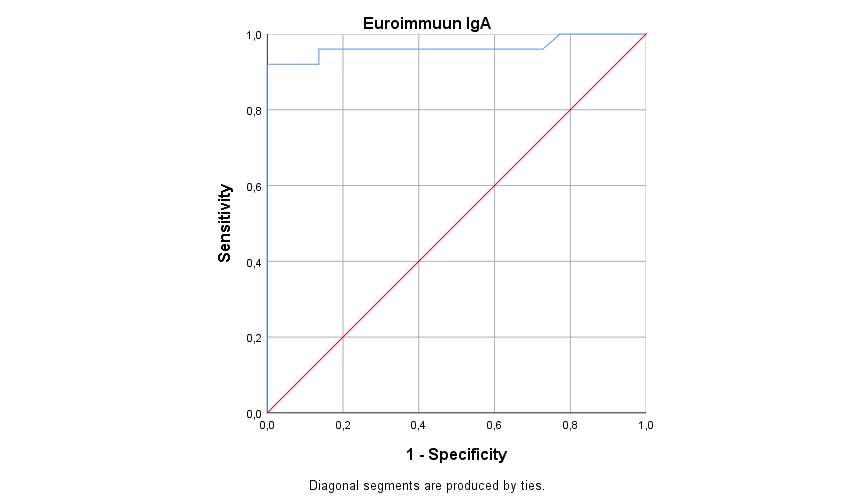

Supplement: Supplementary file 6 — (PNG 14 kb) [file 10096_2021_4220_MOESM6_ESM.png]

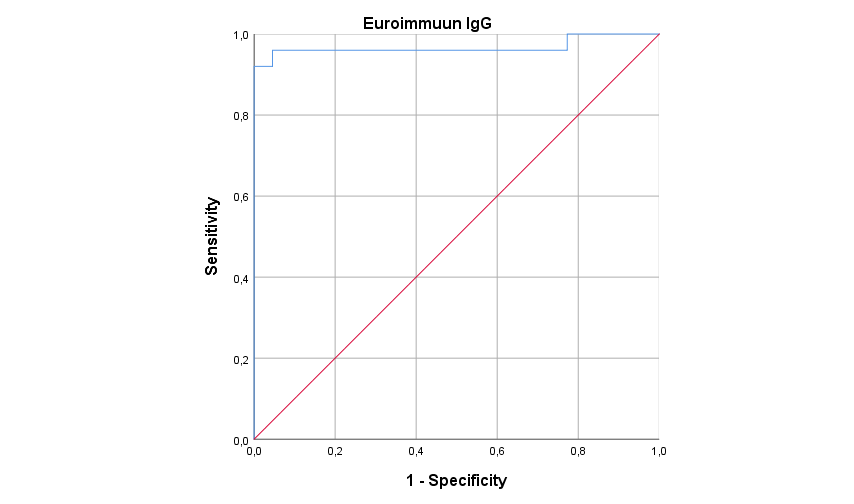

Supplement: Supplementary file 7 — (PNG 12 kb) [file 10096_2021_4220_MOESM7_ESM.png]

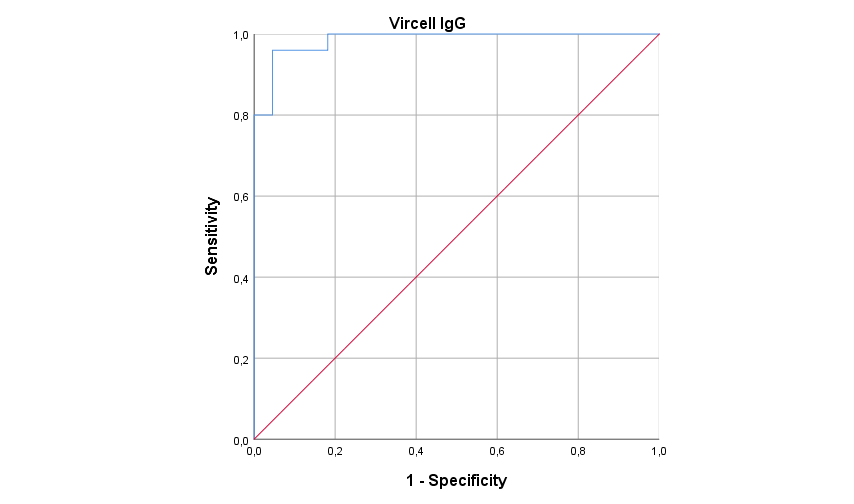

Supplement: Supplementary file 8 — (PNG 12 kb) [file 10096_2021_4220_MOESM8_ESM.png]

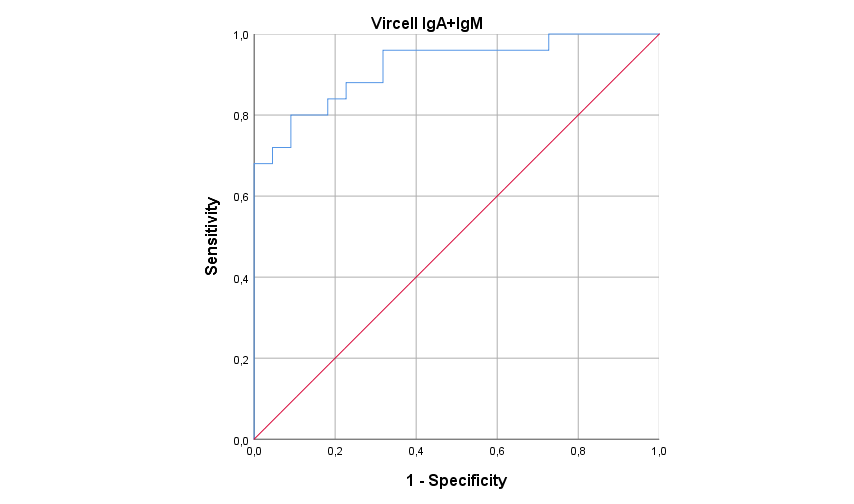

Supplement: Supplementary file 9 — (PNG 12 kb) [file 10096_2021_4220_MOESM9_ESM.png]

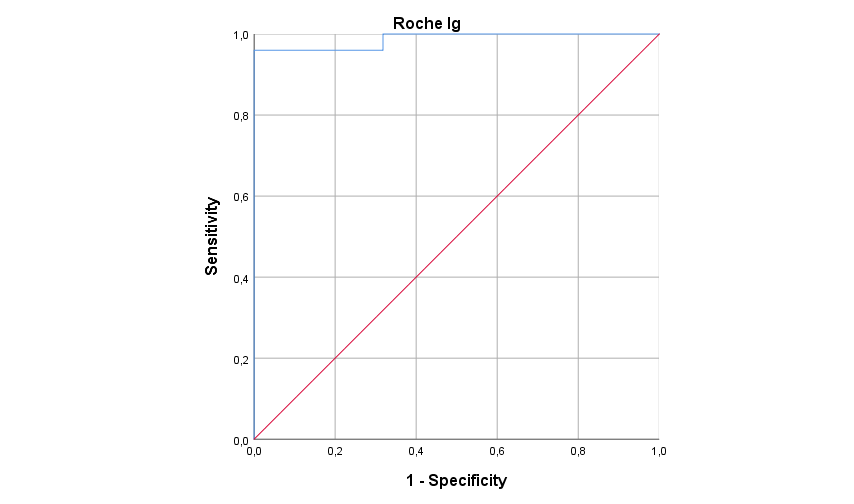

Supplement: Supplementary file 10 — (PNG 12 kb) [file 10096_2021_4220_MOESM10_ESM.png]
